# *Parthenium hysterophorus* alleviates wilt stress in tomato plants caused by *Ralstonia solanacearum* through direct antibacterial effect and indirect upregulation of host resistance

**DOI:** 10.3389/fpls.2023.1126228

**Published:** 2023-02-09

**Authors:** Saba Najeeb, Yan Li

**Affiliations:** State Key Laboratory of Vegetable Biobreeding, Institute of Vegetables and Flowers, Chinese Academy of Agricultural Sciences, Beijing, China

**Keywords:** IDM, phytotoxic, genes, soil, horticulture

## Abstract

Heavy damage to tomato crops due to wilt stress caused by the pathogenic bacterium *Ralstonia solanacearum* and the insufficient availability of management strategies with desired control levels urged the researchers to investigate more reliable control methods to manage this issue in tomato and other horticultural crops. In this study, *Parthenium hysterophorus*, a locally and freely available herbaceous plant, was successfully used to manage bacterial wilt of tomatoes. The significant growth reduction ability of *P. hysterophorus* leaf extract was recorded in an agar well diffusion test and its ability to severally damage the bacterial cells was confirmed in SEM analysis. In both greenhouse and field trials, soil amended with *P. hysterophorus* leaf powder at 25 g/kg soil was found to effectively suppress the pathogen population in soil and significantly reduce the wilt severity on tomato plants, resulting in increased growth and yield of tomato plants. *P. hysterophorus* leaf powder at concentrations greater than 25 g/kg soil caused phytotoxicity in tomato plants. The results showed that *P. hysterophorus* powder applied through the mixing of soil for a longer period of time before transplanting tomato plants was more effective than mulching application and a shorter period of transplantation. Finally, the indirect effect of *P. hysterophorus* powder in managing bacterial wilt stress was evaluated using expression analysis of two resistance-related genes, *PR2* and *TPX*. The upregulation of these two resistance-related genes was recorded by the soil application of *P. hysterophorus* powder. The findings of this study revealed the direct and indirect action mechanisms of *P. hysterophorus* powder applied to the soil for the management of bacterial wilting stress in tomato plants and provided the basis for including this technique as a safe and effective management strategy in an integrated disease management package.

## Introduction

Phytobacterial diseases cause significant losses by reducing the yield of many important agricultural crops (Obradovic et al., 2008). Among plant pathogenic bacteria, *Ralstonia solanacearum*, the causal agent of wilt disease, is one of the most serious pathogens (Nion and Toyota, 2015). On the basis of geographically varied distribution, *R. solanacearum* forms a species complex consisting of four phylotypes, five races, and six biovars (Prior et al., 2016). This soil-borne disease is widespread worldwide and has a huge host range, encompassing more than 185 species of plants (Vu et al., 2013; Nion and Toyota, 2015). The pathogen produces wilting symptoms, and gradually, plants start drying. Losses caused by *R. solanacearum* to economically important plants vary greatly based on the weather conditions, cultivar and soil types, host susceptibility, etc. Over 15 tomato diseases, including bacterial wilt, have been reported to cause significant damage to tomato crops in several countries worldwide with yield losses ranging from 0% to 96% and yield losses caused by bacterial wilt were reported up to 94.54% (Nion and Toyota, 2015; Khan et al., 2020).

Although several methods have been explored to manage bacterial wilt disease, the majority of the host crops still lack an effective and eco-friendly control technique. Because of the pathogen’s complex nature, growth inside the host, soil persistency, aqueous transport, and biodiversity, it is difficult to control (Nion and Toyota, 2015). Over several decades, various strategies for bacterial wilt disease control have been researched. According to, chemical approaches were primarily discussed in studies of these techniques, followed by biological, cultural, and physical practices. Chemical pesticides have been used extensively to control plant disease. Unfortunately, chemical control has been severely restricted because of its link to chemical resistance, environmental degradation, and toxic side effects (Vidaver, 2002; Montesino, 2007). Because of the rising demand for ecologically friendly pesticides, the search for safe and efficient antimicrobial agents is crucial for treating plant pathogens.

The harmful effects of chemical pesticides can be reduced by using pesticides of plant origin, which contain diverse antimicrobial compounds (Dubey et al., 2011). The use of botanicals as substitutes for synthetic chemical pesticides has grown yearly throughout the world. Particularly, the rise in the production of organic foods has resulted in a significantly higher demand for botanical pesticides in industrialized and developed countries (El-Wakeil, 2013). In several studies, scientists attempted to use plant-based materials to control plant pathogens. The rice seed-borne pathogenic bacteria *Xanthomonas oryzae* was found to be particularly affected by aqueous extracts from the wild medicinal herb *Adhatoda vasica* (Govindappa et al., 2011). Plant material in the form of aqueous extracts and green manures as foliar or seed treatments have already been demonstrated to be effective against different plant diseases (Cardoso et al., 2006; Askarne et al., 2012). However, the organic amendment of soil through plant material, especially *Parthenium hysterophorus*, was not investigated against bacterial wilt disease.

This study aimed to investigate the (i) antibacterial potential of *P. hysterophorus* in detail, (ii) its ability to upregulate host resistance, and (iii) its utilization for the management of tomato bacterial wilt disease in a greenhouse as well as in field conditions. The destructive morphological properties of *P. hysterophorus* against *R. solanacearum* were confirmed through a scanning electron microscope, and upregulation of host resistance was investigated through qRT-PCR analysis of resistance genes in tomatoes. A comprehensive greenhouse and repeated field trials were carried out using *P. hysterophorus* powder as a soil organic amendment for the management of bacterial wilt disease in tomatoes.

## Materials and methods

### Plant and pathogen source

Six plants that were abundantly present along the roadside and in open, uncultivated fields were collected from Muzaffarabad City, Azad Jammu, and Kashmir State, Pakistan (**Supplementary Table S1**). The plants were washed with distilled water and shade-dried for 1 week. Depending on the experimental need, the plants were grounded as a whole or separated into different parts such as roots, stems, leaves, and shoots. The preserved and identified pathogenic strain of *R. solanacearum* was reactivated in Lysogeny broth (LB) medium (peptone 10 g, yeast extract 5 g, sodium chloride 10 g) at 25°C for 48 h, and after confirming the pathogenicity, it was used for different *in vitro* and *in planta* (greenhouse and field) experiments.

### Preparation of plant extract and powder

The finely ground powder of plant or plant parts was extracted with ethanol. Briefly, 50 g of powder was soaked in 200 ml of ethanol for 48 h with continuous stirring, followed by filtration by using a muslin sheet and centrifugation at 8000 rpm for 5 min. Using Whatman filter paper No. 31, a clear filtrate was obtained and was dried under reduced pressure using a vacuum evaporator. The obtained extract was checked for antibacterial activity. For soil amendment, leaves were dried and ground into a fine powder.

### Evaluation of antibacterial activity

An ethanol extract from plants was tested for their bioactivity against *R. solanacearum* through the good diffusion method. The dried extract was dissolved in ethanol at 400 mg/ml. In a Petri plate, 25 ml of LB medium with a bacterial concentration of 10^5^ CFU/ml was poured and allowed to cool for 4–5 min. Eight wells of 3 mm in size were punched through a sharp plastic borer in the medium, and six wells were filled with 10 µl of plant extracts; one well was filled with streptomycin, and one was filled with ethanol as a negative control. After incubating the plates at 25°C for 24 h, the diameter of the inhibition zone around the wells was measured in millimeters. In the next step, the extract of different parts (roots, leaves, stems, and shoots) of the most active plant was tested by using the above method. The most active plant part (leaves) was then tested in different concentrations (100, 200, 300, and 400 mg/ml) to evaluate the concentration-dependent activity of plant extract.

### Phytotoxicity test

To test the phytotoxicity effect of the most active plant part powder, tomato plants were grown in soil amended with different rates of plant powder (5, 10, 15, 20, 25, 30, and 35 g) in 1 kg soil in a pot experiment. Briefly, 20-day-old tomato seedlings (Rio Grande) were transplanted in 1 kg of potted soil with different rates of plant powder at one plant per pot. The plants were grown for 30 days after transplantation with routine horticultural practices. After 30 days, the phytotoxic effect was evaluated by recording data on plant length, plant weight, and the number of leaves per plant. Seven plants were maintained for each treatment, and the experiment was repeated once.

### Greenhouse test

In the greenhouse test, different times (0, 5, 10, and 15 days before transplantation) and methods (mixing and mulching) of *P. hysterophorus* powder at 25 g/kg soil were evaluated for their effect on the soil population of the pathogen, disease severity, and growth and yield of tomato plants. *P. hysterophorus* powder, 25 g, was applied to 1 kg of soil in pots either through mixing or simple mulching (spreading powder over soil) at 0, 5, 10, and 15 days before transplantation. A bacterial suspension of 35 ml of *R. solanacearum* (10^5^ cfu/ml) was poured at the center of each pot. Tomato seedlings were transplanted 0, 5, 10, and 15 days after *P. hysterophorus* powder application in each pot. The plants were grown for 70 days, and data were taken on growth (plant and root length, plant weight) and yield (number of tomato/plant) parameters.

### Disease severity and pathogen population in soil

The disease severity data were recorded over the season up to four times at an interval of 15 days using a disease index rating scale (Wai et al., 2013), and the calculated disease severity value was converted to Area Under Disease Progressive Curve (AUDPC) value by using the method described by Madden et al. (2007). The pathogen population in soil under the influence of different treatments was counted using the serial dilution method. Soil from each pot in the rhizosphere region was sampled using a 12-cm-long cork borer. *R. solanacearum*, a pathogenic bacteria, was isolated from soil samples on selective growth medium tetrazodium chloride nutrient agar (TZCNA). Isolated bacterial colonies were counted, and the data were converted to log_10_. From each pot, three independent samples were taken and used as subreplicate for each treatment.

### Field test

This experiment was designed to test the field efficacy of *P. hysterophorus* powder against bacterial wilt disease in tomato plants. The experimental field area was equally divided into three sets of plots and each set contained two plots, each 8 m^2^ in size. Each plot consisted of three lines and each line was transplanted with 12 tomato plants. The 100-ml pathogen inoculum (10^5^ cfu/ml) was mixed with the soil by pouring in holes in two sets of plots followed by amendment of this soil with *P. hysterophorus* powder through mixing method, fifteen days prior to tomato (Rio Grande) transplantation in one set (T1: P+P) and the other set with only pathogen inoculation (T2: P). Both pathogen inoculum and *P. hysterophorus* powder were applied to the soil around the point of the plantation. The third set of plots neither received pathogen inoculum nor *P. hysterophorus* powder kept as control (C). The plants were grown for 70 days, and data on soil bacterial pathogens, disease severity, and plant growth and yield were recorded as above in the greenhouse experiment.

### Evaluation of resistance induction

Tomato seedlings (20 days old) were grown in soil mixed with *P. hysterophorus* leaf powder in two groups. Plants in one group were kept uninoculated, while in the other group, they were inoculated with *R. solanacearum* suspension (1 × 10^7^). Control plants were maintained by using sterile water treatment. After inoculation of the pathogen, at 0, 6, 12, 24, 48, and 72 h, the leaves were collected for RNA analysis. TRNzol Universal reagent was used for the extraction of total RNA FastKing RT Kit was used for reverse transcription of 1 µg of total RNA after DNase treatment. PCR was performed in a 25-µl buffer reaction. The composition of the PCR mixture and running conditions are presented in **Supplementary Table S2**. The expression of *PR2* and *TPX* marker genes was analyzed using primers F-5′GGACACCCTTCCGCTACTCTT3′; R-5′TGTTCCTGCCCCTCCTTTC3′ and F-5′GAGATGCAGTTGTGGCTACG3′; R-5′GCGAAGGATTGTTGCAGTCT3′, respectively, in the leaf samples, and actin was kept as internal reference gene. The experiment was performed in triplicates.

### Statistical analysis

*In vitro* experiments were conducted using the CRD design, while for field evaluation, RCBD was maintained. The data on plant yield and growth, pathogen population in the soil, and severity of the disease were analyzed as dependent variables, while the methods and duration of applications were analyzed as independent variables using the Statistix 8.1 package. The significant treatment mean was differentiated using the LSD test *p* = 0.05.

## Results

### *Antibacterial activity of different medicinal plant extracts against* R. solanacearum

**Figure 1:**
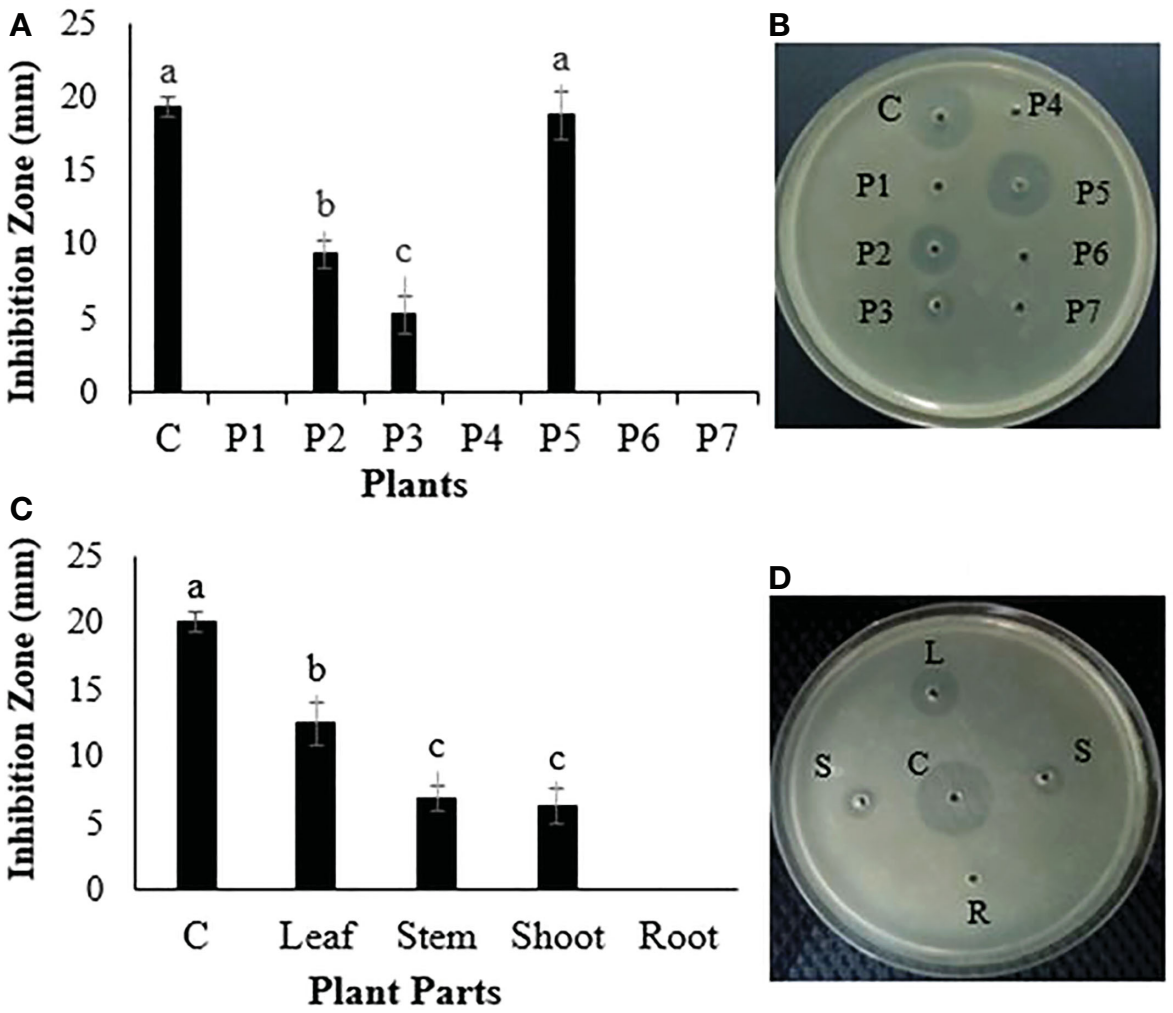
**(A, B)** Antibacterial evaluation of different plants against a bacterial wilt pathogen. **(C, D)** Antibacterial evaluation of different parts of *P. hysterophorus* against a bacterial wilt pathogen. P1, *Broussonetia papyrifera*; P2, *Dodonaea viscosa*; P3, *Peganum harmala*; P4, *Parthenium hysterophorus*; P5, *Mentha piperita*; P6, *Fumaria parviflora*. Different lowercase letters show significant difference among treatments.

The ethanol extracts of different medicinal plants were tested for *in vitro* bacterial growth inhibition against *R. solanacearum*. Three plant extracts, *Dodonaea viscosa*, *Peganum harmala*, and *P. hysterophorus*, inhibited the growth of *R. solanacearum*, with the maximum growth inhibition of *R. solanacearum* being recorded by the *P. hysterophorus* extract. The inhibition zone given by *P. hysterophorus* extract was 18.7 ± 0.7 mm, which was nearly similar to the inhibition zone produced by the positive control streptomycin at 19.3 ± 1.2 mm. The growth inhibition zones recorded for *Dodonaea viscosa* and *Peganum harmala* were 9.3 ± 1.1 mm and 5.2 ± 0.6 mm, respectively. The extracts of other plants did not show any activity (**Figures 1A, B**).

After the screening of several plants, different parts of the most active plant (*P. hysterophorus*) were tested against *R. solanacearum in vitro* growth inhibition. Among different plant parts, leaf extract inhibited maximum *in vitro* growth of *R. solanacearum* by giving the biggest zone of inhibition (13.5 ± 0.8 mm), followed by shoot and stem extract, while root extract did not show antibacterial activity (**Figures 1C, D**).

### Concentration-dependent antibacterial activity and cellular destruction

**Figure 2:**
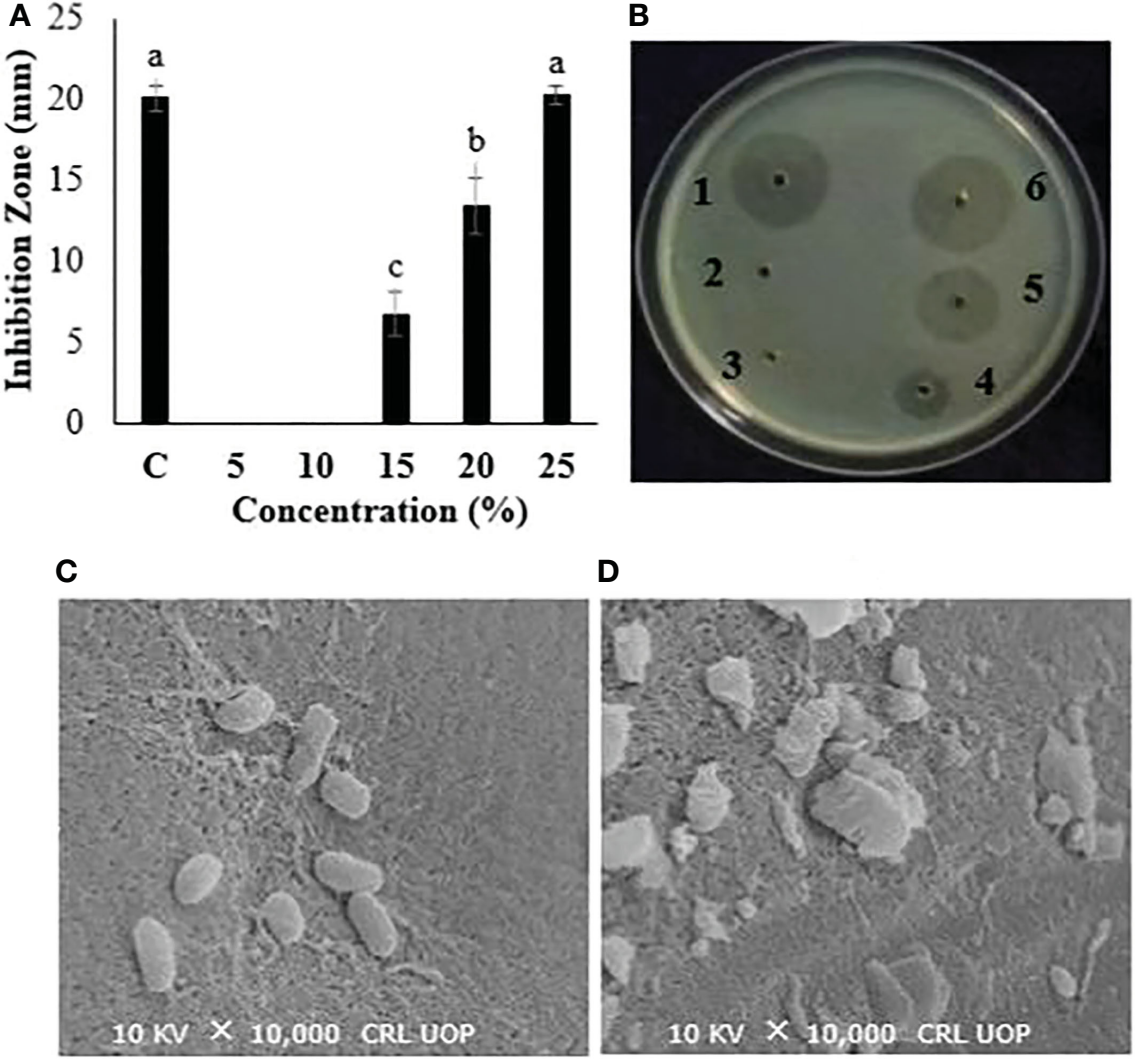
**(A, B)** Antibacterial evaluation of *P. hysterophorus* leaf extract in various concentrations. B1, streptomycin; B2, ethanol; B3, 100; B4, 200; B5, 300; and B6, 400 mg/ml against a bacterial wilt pathogen. **(C, D)** SEM figures of bacterial cells. Different lowercase letters show significant difference among treatments.

Different concentrations (100, 200, 300, and 400 mg/ml) of the *P. hysterophorus* leaf extract were tested to investigate the concentration-dependent antibacterial activity. Results showed that increases in the concentration of the extract resulted in increased antibacterial activity. The lowest concentration, 100 mg/ml, was not active. The concentrations of 200, 300, and 400 mg/ml showed significant inhibition of bacterial growth by giving 6.8, 13.8, and 19.6 mm inhibition zones, respectively. The zone of inhibition produced by the highest concentrations is nearly equal to the one produced by positive control at 20.1 mm (**Figures 2A, B**). Bacterial cells from the biggest inhibition zone were subjected to SEM analysis to observe the cellular destructions caused by plant extract. Results from SEM images clearly indicated the morphological destructions of bacterial cells treated with leaf extract. Compared to untreated cells, the cells treated with plant extract showed swollen and ruptured cellular morphology (**Figures 2C, D**).

### Phytotoxicity test

**Figure 3:**
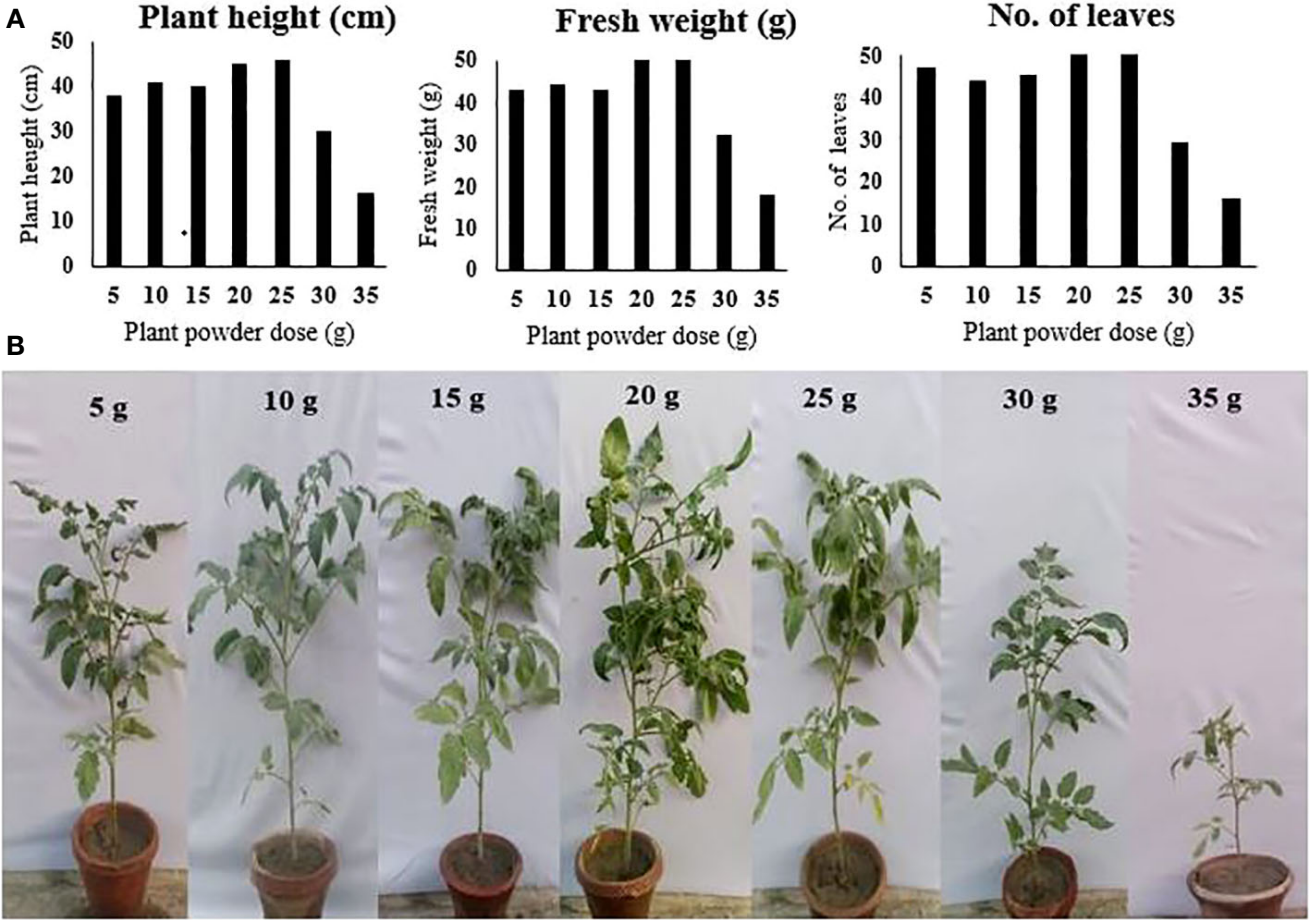
**(A)** Tomato plant growth parameters under the influence of different amounts of *P. hysterophorus* leaf powder. **(B)** Pictures of tomato plants grown in soil amended with different amounts of *P. hysterophorus* leaf powder.

The phytotoxicity of *P. hysterophorus* leaf powder to tomato plants was checked by evaluating the growth parameters of tomato plants sown in soil that was amended with different application rates of *P. hysterophorus* leaf powder. Compared to the control, the treatment in which the soil was amended with plant powder increased plant growth. Although the lower application rates showed a slight increase in plant growth, their effect was statistically not different from the control. The application rate of 25 g/kg soil, however, showed a significantly higher plant length of 46.6 cm, a weight of 53.4 g, and a leaf number of 59.4 as compared to the control, where 37.4 cm, 43.7 g, and 47.2 plant length, weight, and leaf number were recorded, respectively. The higher two doses showed phytotoxicity to tomato plants and caused a significant reduction in plant growth. The highest application rate of 35 g/kg soil showed the lowest plant length of 16.0 cm, a minimum weight of 18.7, and a leaf number of 16.4 (**Figure 3**).

### Greenhouse and field study

#### Pathogen population in soil

**Figure 4:**
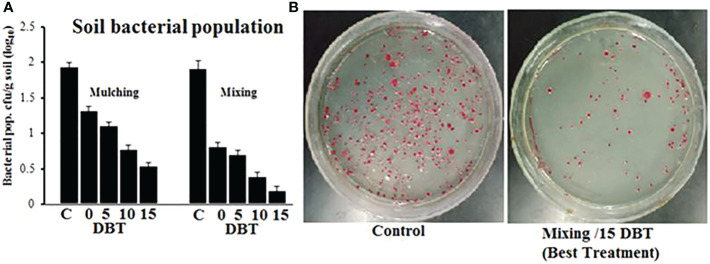
**(A)** Pathogen population in soil amended with *P. hysterophorus* leaf powder at different time durations through mulching or mixing method. **(B)** Comparison CFU of *R. solanacearum* isolated from untreated (control) soil or soil treated with the best treatment.

The soil was amended with *P. hysterophorus* leaf powder through mixing and mulching methods at 0, 5, 10, and 15 days before transplanting. At the end of the experiment, the pathogen population in the soil was quantified through the serial dilution technique. In general, the soil bacterial population decreased as application time increased, and plant powder applied through the mixing method had significantly less pathogen population in the soil than mulching. The plant powder, when applied 15 DBT through the mulching method, showed a 0.55-log_10_ cfu/g soil pathogen population as compared to unamended control soil with a 1.95-log_10_ cfu/g soil pathogen population, while in the case of the mixing method, the same treatment showed lowest soil pathogen population 0.21 log_10_ cfu/g of soil (**Figures 4A, B**).

#### Disease severity

**Figure 5:**
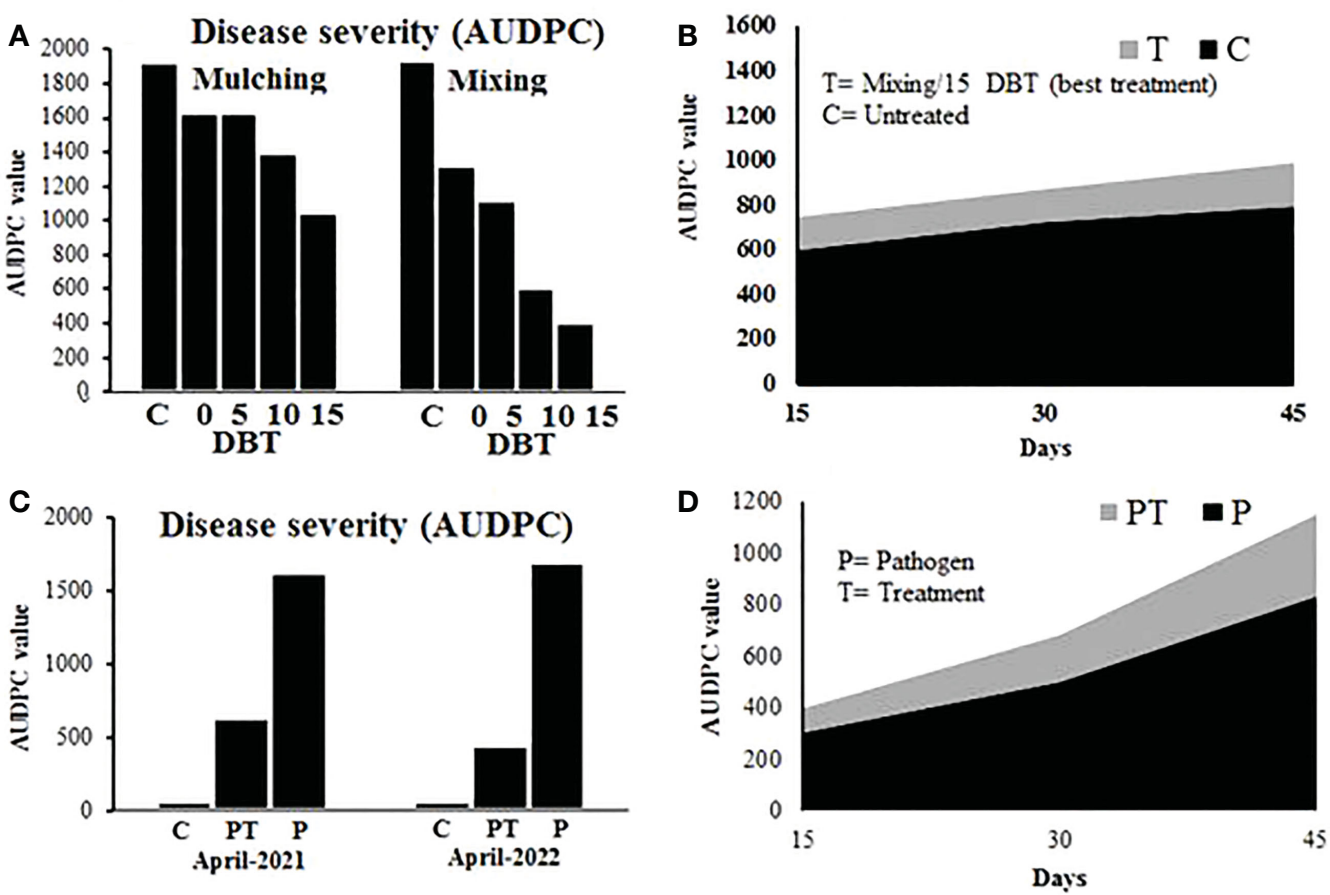
**(A, B)** Disease severity (converted to AUDPC value) on tomato plants grown in soil amended with *P. hysterophorus* leaf powder at different time durations through mulching or mixing methods in a greenhouse trial. **(C, D)** Disease severity (converted to AUDPC value) on tomato plants grown under different treatments in field trials. C, control plants; PT, plants inoculated with the pathogen and treated with *P. hysterophorus* leaf powder; P, untreated plants inoculated with pathogens.

The calculated disease severity was converted to AUDPC value. Results showed that the method and time of plant powder application significantly affected the disease severity of tomato plants. Consistent with the effect on the pathogen population in the soil, the disease severity reduced with an increase in application time, and plant powder applied through the mixing method caused more reduction than simple mulching over the soil. The minimum AUDPC value of 403 was recorded for the plants sown in soil amended with plant powder at 15 DBT through the mixing method, while the corresponding AUDPC value for control in the mixing experiment was 1,940. In the case of the mulching experiment, the AUDPC values for 15 DBT and control treatments were 1,046 and 1,923, respectively (**Figures 5A, B**).

In field tests, the disease severity of pathogen-inoculated plants with or without treatment of plant powder was recorded. Results showed that treatment of tomato plants with plant powder 15 days before transplantation through the mixing method exhibited a significantly lower AUDPC value (630) than the AUDPC value (1626) of untreated inoculated plants. The control plants that were not inoculated with the pathogen did not show disease symptoms (**Figures 5C, D**).

#### Plant growth and yield

**Figure 6:**
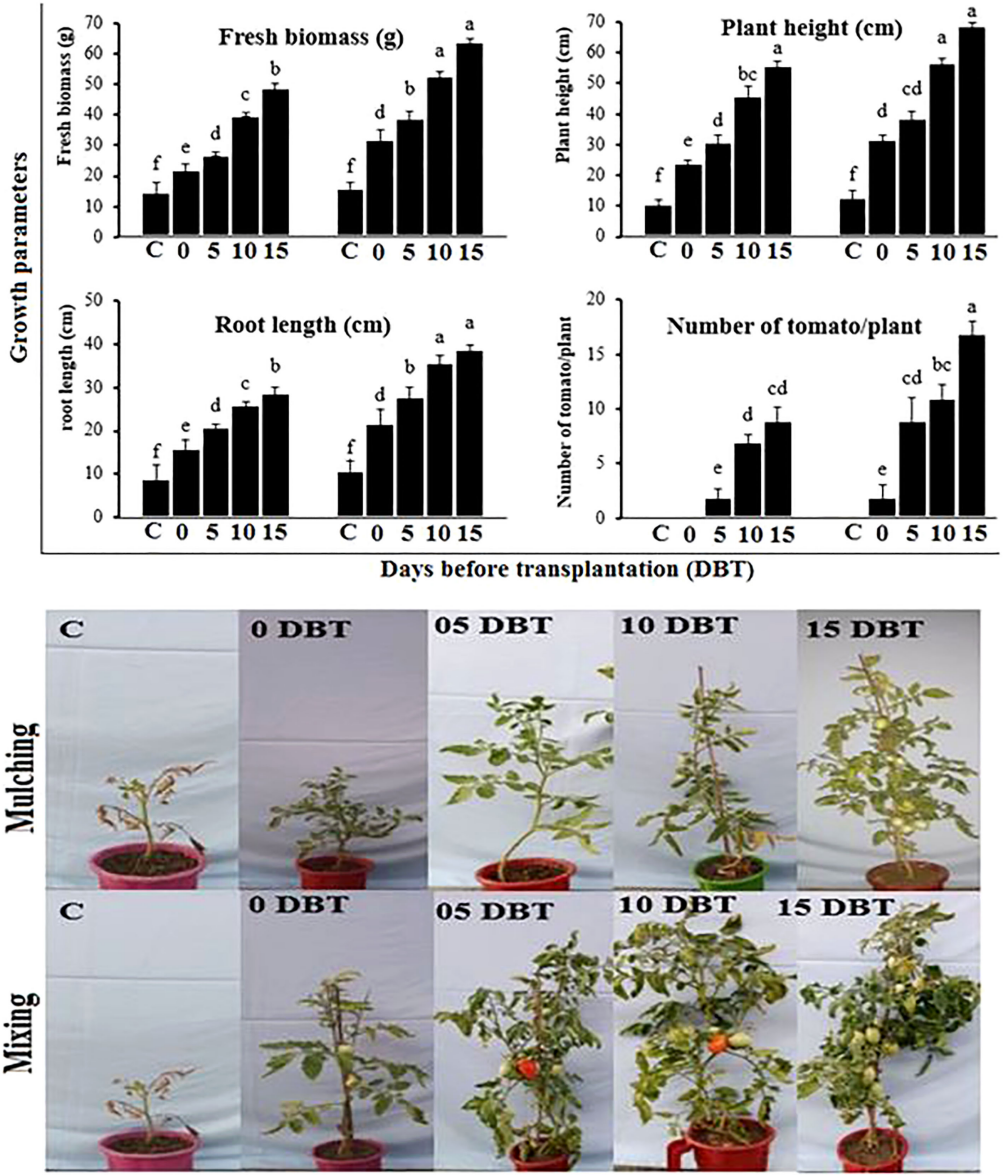
Growth and yield of tomato plants grown in soil amended with *P. hysterophorus* leaf powder at different time durations through mulching or mixing methods in the greenhouse. Different lowercase letters show significant difference among treatments.

Tomato plant height, root length, fresh biomass, and yield were evaluated under the treatment of plant powder applied through mixing or mulching at different application times. Among different application times, the longest time, 15 DBT, was superior over other application times, and the powder applied through the mixing method was batter than the mulching method in terms of improving plant growth and yield under bacterial wilt disease stress (**Figure 6**). The maximum weight of the plant was 64.8 g, the length of the shoot was 69.2 cm, the root was 39.3, and the number of tomatoes per plant was 17.4, shown by the plants sown in soil amended with plant powder at 15 DBT through mixing method. The plant powder when applied with the same application time i.e. 15 DBT but through simple mulching over soil showed 49.2 g, 56.4 cm, 29.7 cm, and 9.3, weight, plant length, root length, and number of tomatoes per plant, respectively. The minimum plant growth and number of tomatoes per plant were shown by the plants sown in the control soil.

**Figure 7:**
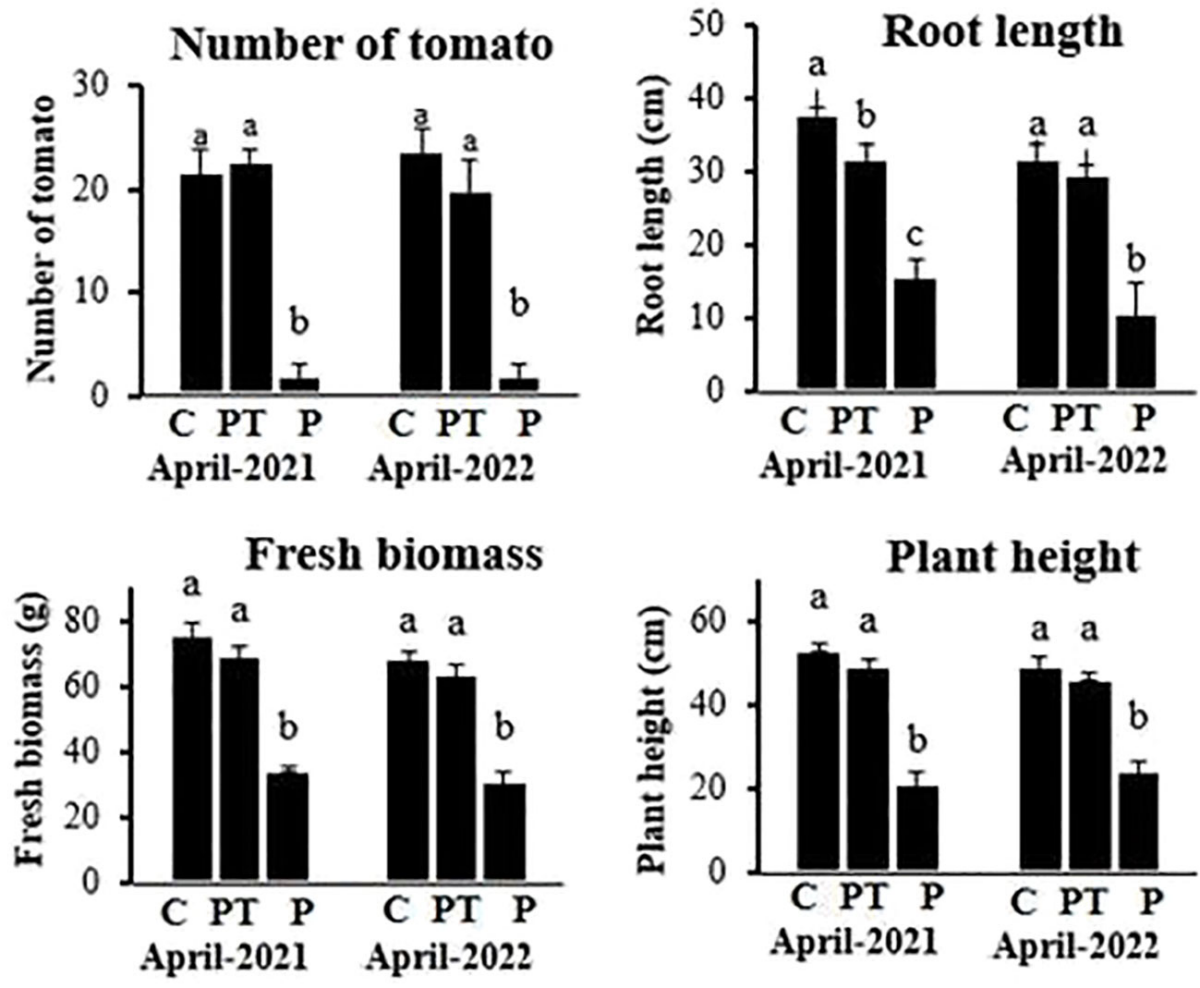
Growth and yield of tomato plants grown under different treatments in field trials. C, control plants; PT, plants inoculated with the pathogen and treated with *P. hysterophorus* leaf powder; P, untreated plants inoculated with pathogens. Different lowercase letters show significant difference among treatments.

In field trials, the pathogen-inoculated plants when treated with plant powder showed significantly higher growth (plant length: 50.4 cm, weight: 70.3 g, and root length: 32.4 cm) and yield (23.7 tomato/plant) compared to untreated inoculated plants (plant length: 22.5 cm, weight: 35.5 g, and root length: 16.7 cm) and yield (2.3 tomato/plant). The uninoculated and untreated control plants showed similar growth and yield as shown by plants treated with plant powder (**Figure 7**).

#### Resistance induction

**Figure 8:**
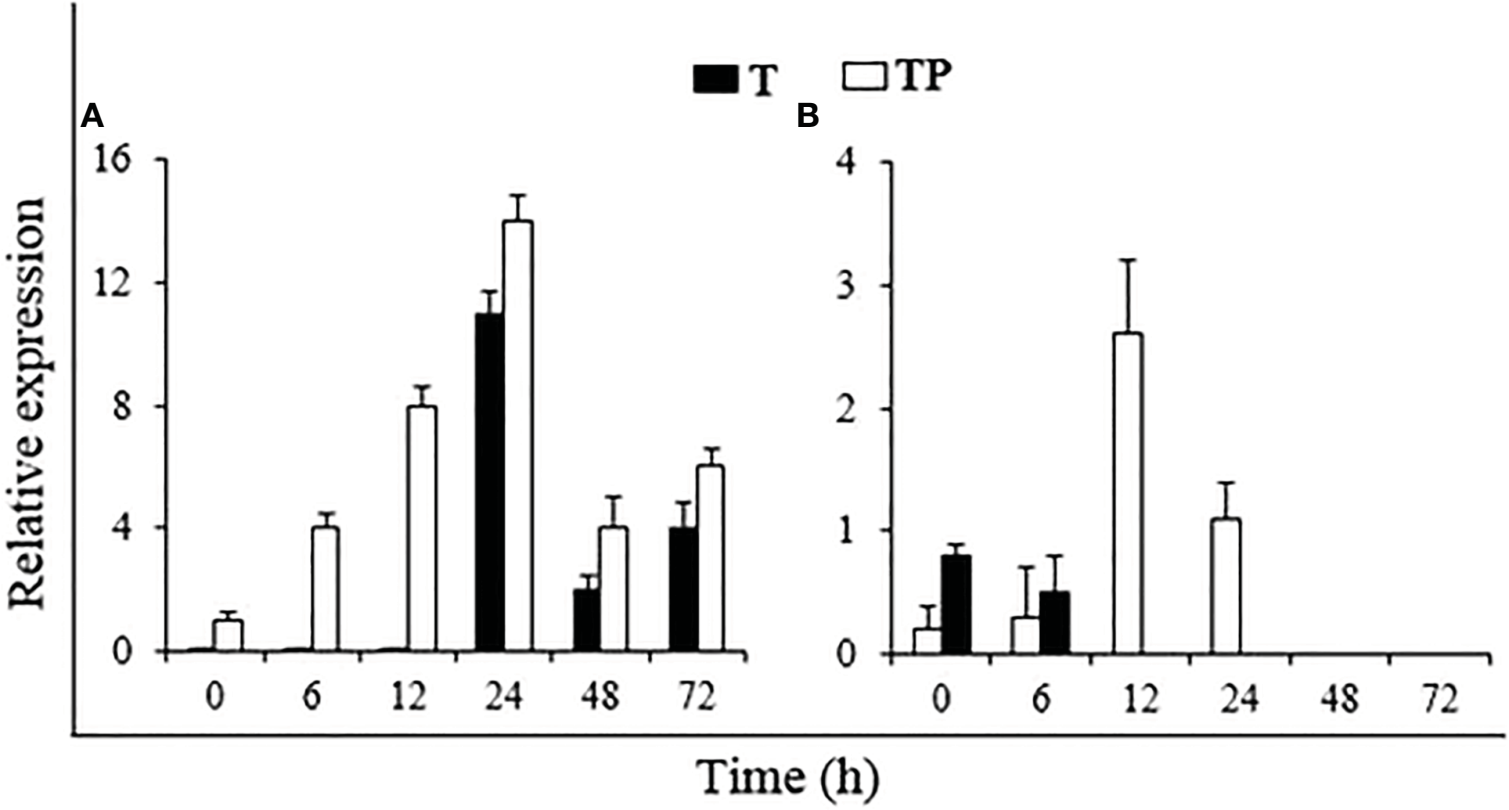
Relative expression of resistance genes (**A**: *PR2* and **B**: *TPX*) in tomato plants under different treatments. T: Un-inoculated plants grown in soil amended with *P. hysterophorus* powder, TP: Plants treated with *P. hysterophorus* powder and inoculated with *R. solanacearum*.

To test whether the application of *P. hysterophorus* powder induces host resistance to *R. solanacearum*, the expression of the resistance-related genes *PR2* and *TPX* was measured through qRT-PCR analysis. The expression pattern of these two genes in plant powder-treated plants inoculated with or without the pathogen was investigated after 0, 6, 12, 24, 48, and 72 h of treatment (**Figure 8**). In uninoculated plants, the expression of *PR2* was initiated after 24 h of plant powder treatment and then decreased. In pathogen-inoculated plants, the *PR2* gene started expressing from the start, peaked at 24 h, and then decreased. The *TPX* gene was expressed from the beginning in both uninoculated and inoculated plants. However, in uninoculated plants, the *TPX* expression stopped after 6 h, but in pathogen-inoculated plants, it showed higher expression until 24 h, with a peak at 12 h.

## Discussion

The tomato, a nutrition-rich plant with a high source of income for farmers, is one of the most widely grown plants worldwide. However, various tomato plant diseases affect the products in terms of quantity and quality, and therefore, decrease productivity. Bacterial wilt caused by *R. solanacearum* is one of the well-known diseases of tomatoes that cause severe destruction not only in tomato crops but in a wide range of economically important host plants. Management of plant diseases greatly relies on chemicals, but the harmful effects of these synthetic chemicals, along with the pathogen’s potential to develop resistance, have urged scientists to investigate chemical-free strategies for the management of plant pathogens (Dasgupta et al., 2007; Mamphogoro et al., 2020). Because of this circumstance, scientists in different countries are becoming more interested in using organic amendments (OAs) and green manures to reduce plant diseases (Scotti et al., 2015).

Numerous researchers have reported the effectiveness of plant-based OAs against various phytopathogens (Bonanomi et al., 2007), such as fungi (Kareem et al., 2008), nematodes (Al-Shaibani et al., 2008), bacteria (Ranjit et al., 2012), as well as viral pathogens (Khan et al., 2001). The population of plant parasitic nematodes in the soil was significantly decreased by amending the soil with the dried powder of *Fumaria parviflora* (Naz et al., 2015). In another study, successful suppression of Sarpagandha leaf spot disease was achieved through organic amendments of *Pongamia pinnata* and *Millettia indica* as a seed cake (Arumugam et al., 2010). Tomato bacterial wilt was effectively suppressed by using different parts of *Cajanus cajan* and *Crotalaria juncea* as green manure (Cardoso et al., 2006). In this study, we tested various medicinal plants that abundantly grow in open uncultivated fields or roadsides for their potential to manage bacterial wilt of tomatoes.

Among six plants tested in this study, the extract of *P. hysterophorus* showed the highest antibacterial activity. Evaluation of different parts of *P. hysterophorus* revealed that leaf extract possesses higher activity than other plant parts in a concentration-dependent way. The highest concentration, 400 mg/ml, exhibited a maximum growth inhibition zone compared to other lower concentrations. The inhibition of bacterial growth by extracts of medicinal plants is attributed to the natural bioactive compounds present in them. The superiority of leaves over other plant parts can be explained on the basis of a higher amount of antibacterial substances present in leaves. Generally, plants producing antimicrobial compounds contain these compounds in leaves. According to reports, the leaves of the medicinal plant *Withania somnifera* have higher concentrations of antimicrobial compounds than the rest of the plant (Singh and Kumar, 2011). The morphology of bacterial cells treated with *P. hysterophorus* leaf extract was studied through SEM analysis. Severe morphological destructions to bacterial cells were obvious in SEM images compared to the normal morphology of cells in the control group. The destruction of bacterial cell morphology is due to the membrane disruption properties of antibacterial chemical compounds present in the leaf extract. Phytochemicals were previously demonstrated to disrupt or rupture the bacterial cell membranes, which resulted in abnormal cell morphology observed in SEM analysis (Kamonwannasit et al., 2013).

The *P. hysterophorus* leaves powder was selected for soil amendment for the control of bacterial wilt disease in tomato plants. Before using *P. hysterophorus* leaf powder, the maximum safe amount for organic soil amendment to manage bacterial wilt disease was confirmed by the phytotoxicity test. Results showed that *P. hysterophorus* powder applied at 25 g/kg soil was safe as plants grown in soil amended with a rate higher than 25 g/kg exhibited a significant reduction in growth. There have been reports of using dry powders at a maximum dose of 45g/kg of soil and green manures at a maximum dose of 55g/kg of soil without experiencing any observable phytotoxic effects (Flores-Moctezuma et al., 2006; Cavoski et al., 2012). However, depending on the plant type to be protected and the type of plant used to make an organic amendment, phytotoxic levels may differ from plant to plant. In this study, the higher two amounts (30 and 35 g/kg soil) caused a significant reduction in plant growth that suggests phytotoxicity. Therefore, before using such powders on a commercial scale, it is necessary to find out the phytotoxic threshold level for each plant powder and each target crop.

The maximum safe amount of *P. hysterophorus* powder was then evaluated as a soil amendment to control the bacterial wilt of tomatoes through different times and methods of applications in greenhouse trials. Among different times and methods of application, the soil amendment with *P. hysterophorus* powder 15 days before transplantation through the mixing method achieved higher suppression of pathogen population in the soil, enhanced plant growth and yield, and reduction in disease severity. The optimal application timing of 15 DBT might be explained better by the fact that there was more time for the plant powders to break down and release antibacterial compounds and that *R. solanacearum* was exposed to these compounds for a longer period of time. This resulted in a higher suppression of the pathogen population in soil, which leads to a decrease in disease severity and an enhancement in plant growth and yield. Similar results were obtained by Aliyu et al. (2010), who reported increased yield and growth of cowpea by applying *Azadirachta indica* leaf powder at 14 DBT. The maximum safe amount of *P. hysterophorus* powder was then evaluated for managing bacterial wilt of tomato in field conditions by using the best application time 15 days before transplantation and method of application (mixing). Consistent with greenhouse results, the artificially inoculated field soil mixed with 25 g *P. hysterophorus* leaf powder per plant rhizosphere before 15 days of tomato transplantation significantly reduced bacterial wilt disease on tomato plants. This treatment caused higher growth of plant and tomato yield as compared to plants in the control group.

The use of plant materials as a soil amendment for the management of plant diseases has been previously reported in several studies (Naz et al., 2015). Plant residue integrated into the soil has a variety of effects. The discharge of bioactive compounds is the most visible effect of the breakdown of organic matter in the soil, especially at high temperatures that accelerate the release of such antimicrobial molecules (Bonanomi et al., 2007). These antimicrobial compounds can damage the pathogen directly (Regnault-Roger et al., 2005). Dried plant powders were also reported to have compounds that act as elicitors for plant defense to upregulate the host resistance against pathogens (Walters et al., 2005). Various plant extracts have been shown to act as natural SAR elicitors in addition to having direct antimicrobial effects, as demonstrated by the *in vitro* reduction of pathogen growth (Kagale et al., 2004; Hassan et al., 2009; Mitra and Paul, 2017). Consistent with the results reported in these studies, we also found the antibacterial and host resistance upregulation activities of *P. hysterophorus* powder. The resistance-related genes *PR2* and *TPX* investigated in this study were significantly upregulated in the leaves of tomato plants grown in soil amended with *P. hysterophorus* powder.

Considering its strong *in vitro* antibacterial activity and the *in planta* disease suppression effect of *P. hysterophorus* leaf powder, it has the potential to be a useful part of integrated disease management against bacterial wilt disease in tomatoes. The dried powder also has advantages such as stable shelf life, easy availability, and ease of application. Additionally, as compared to bulky plants, the plant powder could be conveniently carried to other areas where it is not locally grown. The nature of purely natural phytoproduct makes it eco-friendly and difficult for pathogens to build resistance. According to reports, OA is efficient, nontoxic, and easily biodegradable. Using plant-based products to manage plant diseases is cost-effective, especially if these plants are abundantly grown on unwanted land. These characteristics make the use of such plants an appealing part of integrated disease management for resource-constrained farmers in developing or underdeveloped countries.

## Conclusion

The significant growth reduction ability of *P. hysterophorus* leaf extract was recorded in an *in vitro* test, and severe damage to bacterial cell morphology was confirmed through SEM analysis. In both greenhouse and field trials, soil amended with *P. hysterophorus* leaf powder at 25 g/kg was found to effectively suppress the pathogen population in soil and significantly reduce the wilt severity on tomato plants, resulting in increased growth and yield of tomato plants. The indirect effect of *P. hysterophorus* powder in managing bacterial wilt stress was evaluated through expression analysis of two resistance-related genes *PR2* and *TPX*. The upregulation of these two resistance-related genes was recorded by the application of *P. hysterophorus* powder. The findings of this study revealed the direct and indirect action mechanisms of *P. hysterophorus* powder applied to the soil for the management of bacterial wilting stress in tomato plants.

## Data availability statement

The original contributions presented in the study are included in the article/**Supplementary Material**. Further inquiries can be directed to the corresponding author.

## Author contributions

SN: investigation and writing—original draft. YL: conceptualization, review, editing, funding, and supervision. All authors contributed to the article and approved the submitted version.
