# Supplementary material for: Parthenium hysterophorus alleviates wilt stress in tomato plants caused by Ralstonia solanacearum through direct antibacterial effect and indirect upregulation of host resistance
Source: Front Plant Sci. 2023 Feb 9;14:1126228. doi: 10.3389/fpls.2023.1126228 (PMC9947561; doi:10.3389/fpls.2023.1126228)
Supplement: Supplementary file 1 [file Table_1.doc]

Table S1: Plants collected for the evaluation of antibacterial activity against R. solanacearum

| S. No | Plant Name | Location |
| --- | --- | --- |
| 1 | *Dadonia viscosa* | Muzaffarabad, AJK, Pakistan |
| 2 | *Broussonetia papyrifera* | Muzaffarabad, AJK, Pakistan |
| 3 | *Peganum harmala* | Muzaffarabad, AJK, Pakistan |
| 4 | *Mentha piperita* | Muzaffarabad, AJK, Pakistan |
| 5 | *Fumaria parviflora* | Muzaffarabad, AJK, Pakistan |
| 6 | *Parthenium hysterophorus* | Muzaffarabad, AJK, Pakistan |

Table S2. Composition of reaction buffer used in PCR for the analysis of defense related genes

| **Substance** | **Amount** |
| --- | --- |
| 2×SuperReal PreMix Plus | 5 μL |
| forward primer | 0.5 μL |
| reverse primer | 0.5 μL |
| cDNA template | 1 μL |
| RNase-Free ddH2O | 3 μL |
